# Effectiveness of remote exercise programs in reducing pain for patients with knee osteoarthritis: A systematic review of randomized trials

**DOI:** 10.1016/j.ocarto.2022.100264

**Published:** 2022-05-14

**Authors:** C.G. McHugh, A.M. Kostic, J.N. Katz, E. Losina

**Affiliations:** a Orthopaedic and Arthritis Center for Outcomes Research, Brigham and Women's Hospital, Boston, MA, USA; b Harvard Medical School, USA; c Department of Biostatistics, Boston University School of Public Health, USA

**Keywords:** Osteoarthritis, Knee, Telemedicine, Exercise, RCT, MCID

## Abstract

**Objective:**

Remote knee osteoarthritis (OA) management programs are becoming more popular. This systematic review examined the efficacy of remote exercise programs for relieving pain in persons with knee OA.

**Design:**

We conducted a search of studies published between January 1st, 2013 to March 31st, 2021 in PubMed, Embase, and MEDLINE. We included randomized trials of patients with knee OA or chronic knee pain, studying interventions with an element of telehealth exercise management, and evaluating knee pain as an outcome. Interventions could include fully remote or both remote and in-person components. We excluded observational cohort studies, pilot studies, and studies with poor Physiotherapy Exercise Database (PEDro) scores. Two reviewers extracted pain data, consisting of mean differences from baseline and between groups, and compared them to minimum clinically important difference (MCID) thresholds.

**Results:**

We identified 1867 reports, of which eleven trials with a total of 1861 participants met inclusion criteria. Only one trial demonstrated a clinically meaningful change from baseline between groups. Four interventions were found to result in clinically meaningful improvements in pain from baseline.

**Conclusion:**

This review was limited by variability in outcome measures, intervention content, and comparators. One trial with an inactive control demonstrated clinically meaningful between group differences in pain. All four interventions demonstrating meaningful improvements from baseline included study-initiated communications to discuss and personalize remotely delivered exercise programs. More studies comparing fully or partially remote exercise programs with both active and inactive controls could help optimize the use of remote programs for management of knee OA pain.

## 1 Introduction

Knee osteoarthritis (OA) affects over 14 million Americans annually [1]. As there are currently no disease modifying drugs available to treat knee OA, symptom management is the main course of treatment. Common treatments include physical therapy (PT), intra-articular corticosteroid injections, and – in advanced cases – total knee replacement [[2], [3], [4], [5]]. Exercise and PT are the most frequently recommended interventions for knee OA patients [3,4,6]. However, PT can be expensive in terms of provider- and transportation-related costs [7]. Especially in the context of the COVID-19 pandemic, there has been increased interest in the feasibility of managing OA symptoms using remote telemedicine exercise or PT programs. A sizable body of evidence points to the effectiveness of in-person exercise programs in knee osteoarthritis pain management, but a consensus has not been reached on the effectiveness of remote exercise programs [2,4,6].

Past reviews of telemedicine in the context of OA have examined the effectiveness of self-management plans. However, they were not focused specifically on knee OA, included interventions with additional therapeutics (i.e., depression treatment, injections), and/or selected only trials with uniform outcome measures to allow for meta-analysis. This affects the generalizability of their conclusions to exercise telemedicine interventions for knee pain, as they excluded interventions that are otherwise pertinent [[8], [9], [10], [11]]. A 2013 review by Pietrzak et al. found that internet-based multi-component management programs for osteoarthritis can result in improvements in key measures of health status [12]. A meta-analysis of RCTs examining internet-based rehabilitation programs for knee osteoarthritis performed in 2021 revealed improvements in pain but not physical function [10]. This meta-analysis however did not include non-internet (e.g. telephone) interventions. It did include cohorts with hip OA and interventions that did not involve exercise. Further, it did not analyze whether results were clinically meaningful [10].

This review includes randomized studies and focuses specifically on clinically relevant reductions in pain, which was reported as the most desired treatment outcome in the Arthritis Foundation's 2017 Voice of the Patient Report [13]. In this review we summarize the impact of remote programs that include combinations of self-managed or provider-guided exercise on chronic knee pain or knee OA symptoms. We determined whether the interventions resulted in clinically meaningful changes from baseline in pain, and in clinically meaningful differences in pain reduction as compared with traditional PT or wait list/education control.

## 2 Methods

We conducted this review according to the Preferred Reporting Items for Systematic Reviews and Meta-Analysis (PRISMA) statement [14]. This search protocol was registered prospectively in the PROSPERO database (CRD42022299541). We performed a PubMed search in November 2021 using terms related to knee osteoarthritis and eHealth (Appendix 1). We excluded animal studies and those published before 2013 using PubMed filters. Searches of Embase and MEDLINE databases were performed in March 2022 using identical search terms (system-specific changes were made only when necessary) and filters (Appendix 1). We included randomized controlled trials (RCTs) designed to test the effectiveness of remote exercise interventions (including online, telephone, SMS, or app-based interventions) on a study population with knee OA or chronic knee pain. Studies that include exercise programs in conjunction with other components (e.g., pain coping, self-effectiveness programs, motivational interviewing) were included to reflect practice guidelines recommending multimodal programs including these measures for knee OA [4]. We excluded pilot studies, studies written in a language other than English, studies that included the use of an additional pharmaceutical intervention, study protocols, studies of subjects who did not have knee OA or chronic knee pain, studies that did not report pain as an outcome, qualitative studies, abstracts, and studies of low methodologic quality (score of less than 6 out of 10) as measured on the Physiotherapy Evidence Database (PEDro) scale. Included studies were published between January 1, 2013–March 31, 2021, as earlier studies do not reflect relevant advances in telemedicine-related technology.

**Fig. 1:**
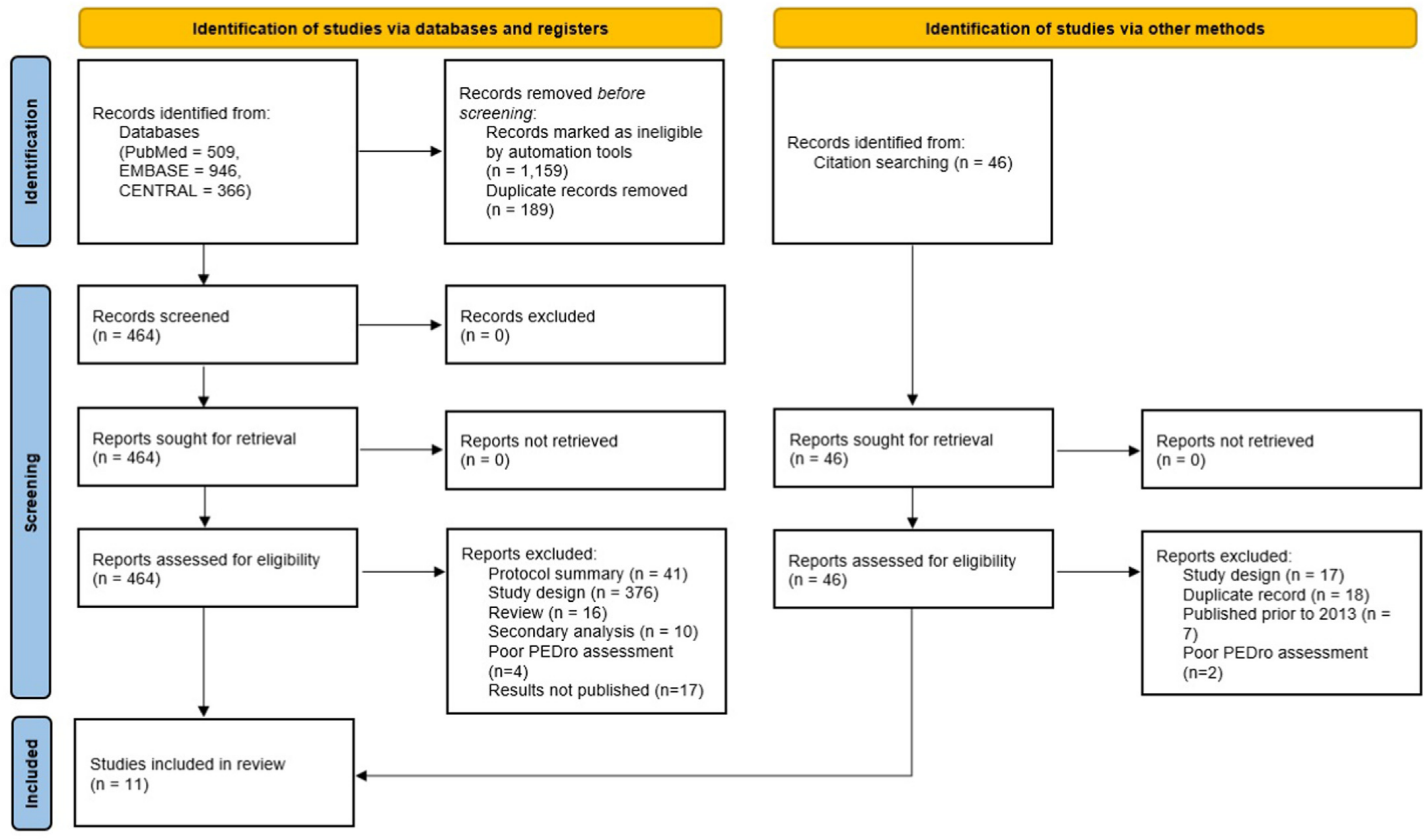
PRISMA flow diagram for screening search results and included studies.

CM and AK each assessed the initial search results independently according to the aforementioned eligibility criteria. We excluded some studies on the basis of their titles, others on the basis of their abstracts, and still others on the basis of the full text (Fig. 1). EL resolved any discrepancies. The two reviewers also independently hand searched reference lists of relevant literature reviews and included studies.

We used the PEDro scale to assess study quality and bias. All included studies had a score available in the PEDro database [[15], [16], [17]]. The PEDro scale evaluates study eligibility criteria, random allocation, concealed allocation, baseline comparability, blinded subjects, blinded therapists, blinded assessors, adequate follow up, intention-to-treat analysis, between-group comparisons, and point estimates and variability. Any studies otherwise eligible for inclusion with a score below 6 out of 10 were excluded on the basis of poor methodological quality [18].

Authors CM and AK extracted results from pain outcome measures independently and resolved disagreements through discussion with the senior author. We recorded participant and intervention characteristics, including sample size (total and in each arm), average age, percent of female subjects, trial design, features of the intervention and control arms, duration of the intervention, all pain outcomes reported, and length of follow-up. CM and AK extracted pain outcomes up to 2 years for each study, including mean changes from baseline in all groups and mean between-group differences in change from baseline at all available timepoints, as well as standard deviations or 95% confidence intervals. For studies that did not report mean change from baseline, we subtracted the mean baseline score from the mean follow-up score. If we were unable to calculate between group differences, we designated them as “not reported.”

Pain outcomes extracted included the Western Ontario and McMaster Universities Osteoarthritis Index pain subscale (WOMAC, 0–20 and 0–100 points), Numerical Rating Scale (NRS, 0–10 points), Visual Analog Scale (VAS, 0–100 points), and Knee Injury and Osteoarthritis Outcome Score pain subscale (KOOS, 0–100 points). Using the minimum clinically important difference (MCID) for each pain outcome as a threshold, as described in Table 1, we defined whether the improvements in pain recorded in each study were clinically meaningful. We used published MCIDs relevant to knee osteoarthritis patients whenever possible [19,20]. The MCID specific to non-surgical treatments for knee OA patients was not available for numerical rating scale pain, and in this case, we used an MCID derived from a patient sample with chronic pain conditions [21].

**Table 1:** Pain outcomes used to measure improvement and the minimum clinically important difference (MCID) in improvement for each.

Pain Outcome	Range	Interpretation	MCID	Studies
Western Ontario and McMaster Universities Osteoarthritis Index (WOMAC) pain subscale	0-20 points or 0–100 points, depending on study	Higher score indicates worse pain	2.4 points on 0–20 scale, 12 points on 0–100 scale [19]	Allen et al. [28]
				Bennell et al. [24]
				Hinman et al. [29]
				Gohir et al. [25]
				Baker et al. [23]
				Bennell et al. [30]
				Allen et al. [22]
				Chen et al. [32]
Numerical rating scale (NRS)	0-10 points	Higher score indicates worse pain	2 points [21]	Bennell et al. [24]
				O'Brien et al. [27]
				Hinman et al. [29]
				Gohir et al. [25]
				Bennell et al. [30]
Visual Analog Scale (VAS)	0-100 points	Higher score indicates worse pain	20 points [20]	Mecklenburg et al. [26]
Knee Injury and Osteoarthritis Outcome Score (KOOS) pain subscale	0-100 points	Lower score indicates worse pain	12 points [19]	Mecklenburg et al. [26]
				Li et al. [33]

For each study we asked two questions: 1) Did the improvement from baseline in the intervention group exceed the improvement from baseline in the comparator group by an extent that met or exceeded the MCID? 2) Did the active intervention group experience improvement from baseline in pain score by an extent that met or exceeded the MCID? The first question was particularly relevant for studies that compared the active intervention to an inactive control such as an education or wait list control. The second question is relevant because participants receive both direct and placebo effects from an intervention, as do patients in practice. To capture the full treatment effect, we examine improvement from baseline.

**Fig. 2:**
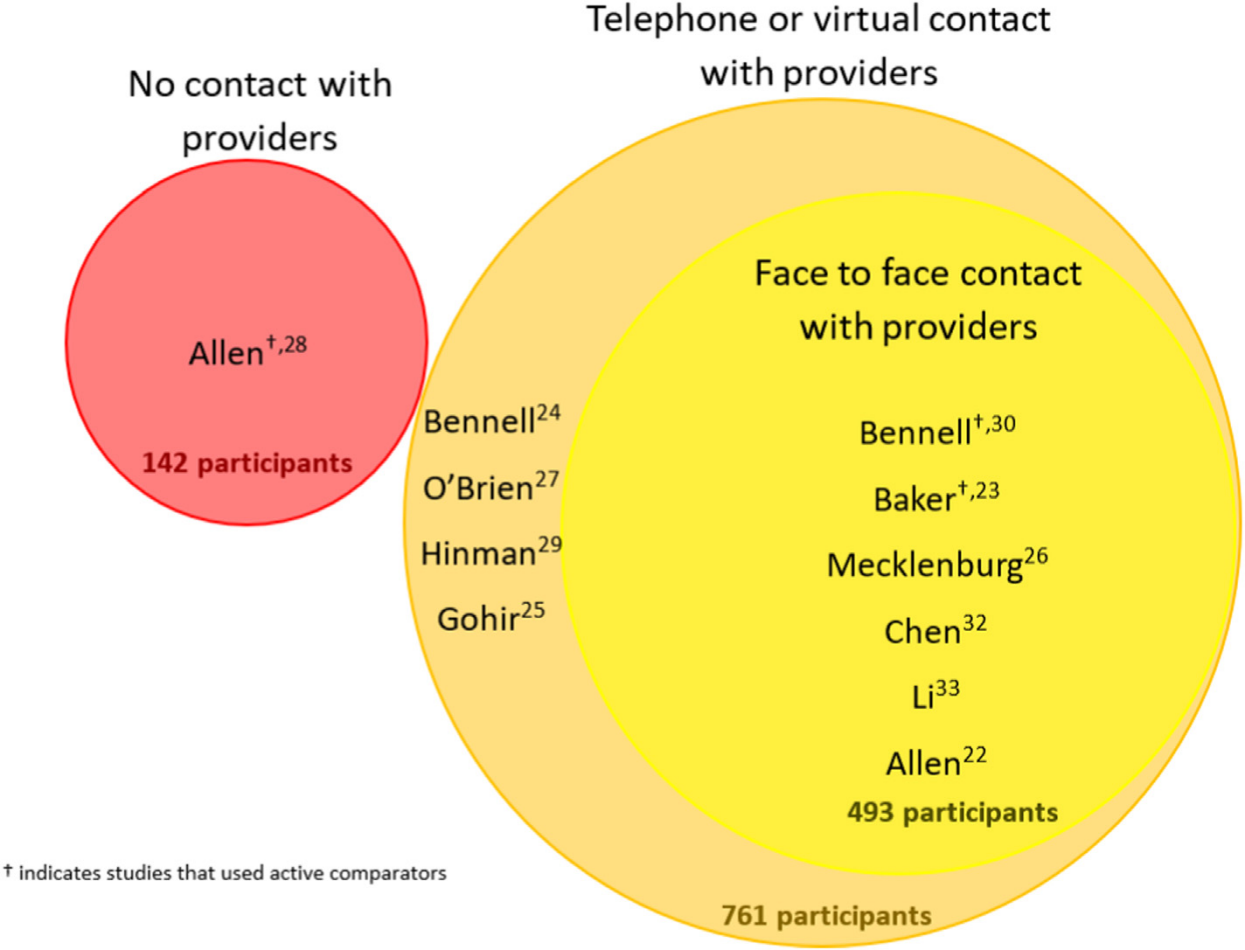
Venn diagram showing the three modalities through which interventions were delivered, with each circle proportional in size to the total number of participants in the intervention arms of the studies in that group. †Indicates studies that used active comparators.

We grouped studies into one of three categories a-priori based on the method of intervention delivery (Fig. 2). The first category included studies whose interventions were delivered through a fully automated process that did not require contact from study staff or providers. The second included those whose interventions were delivered remotely but included some component of telephone or virtual contact with a provider or study staff. The third group comprised hybrid studies that either required or had an option for in-person interactions between clinician and patient. Within each of the three categories, studies are stratified by whether they used an active (e.g., in-person physical therapy) or inactive (e.g., waitlist or education control) comparator and in order of year of publication.

## 3 Results

The initial search yielded 555 results; of these, eleven studies were included in this review (Fig. 1). Of the studies included, one study's intervention was delivered to participants using a fully automated process; 4 studies included some contact from providers over the internet or telephone; and 5 studies included an in-person component.

### 3.1 Quality scoring

All studies included have a PEDro score greater than 6 out of 10 and are considered “good” quality (Table 2) [18]. The greatest risk of bias was found in the way allocation was concealed from participants and assessors. Two studies did not adequately conceal randomization allocation [22,23]. None of the studies adequately blinded subjects or therapists, and three out of the eleven did not adequately blind assessors [[24], [25], [26]]. Three studies did not have adequate follow-up retention [22,25,27].

**Table 2:** Physiotherapy Evidence Database (PEDro) quality assessment scores for included studies.

	Allen [28]	Allen [22]	Baker [23]	Bennell [30]	Bennell [24]	Gohir [25]	Hinman [29]	Li [33]	Chen [32]	Mecklenburg [26]	O'Brien [27]
**Eligibility criteria**	yes	yes	Yes	yes	yes	yes	yes	yes	yes	yes	yes
**Random allocation**	yes	yes	yes	yes	yes	yes	yes	yes	yes	yes	yes
**Concealed allocation**	yes	no	no	yes	yes	yes	yes	yes	yes	yes	yes
**Baseline comparability**	yes	yes	yes	yes	yes	yes	yes	yes	yes	yes	yes
**Blinded subjects**	no	no	no	no	no	no	no	no	no	no	no
**Blinded therapists**	no	no	no	no	no	no	no	no	no	no	no
**Blinded assessors**	yes	yes	yes	yes	no	no	yes	yes	yes	no	yes
**Adequate follow up**	yes	no	yes	yes	yes	no	yes	yes	no	yes	no
**Intention-to-treat analysis**	yes	yes	yes	yes	yes	yes	yes	yes	no	yes	yes
**Between-group comparisons**	yes	yes	yes	yes	yes	yes	yes	yes	yes	yes	yes
**Point estimates and variability**	yes	yes	yes	yes	yes	yes	yes	yes	yes	yes	yes
**Total score**^a^	8	6	7	8	7	6	8	8	6	7	7
**Quality assessment**	Good	Good	Good	Good	Good	Good	Good	Good	Good	Good	Good

a Eligibility criteria did not contribute to total score; yes ​= ​1, no ​= ​0; **highest possible score ​= ​10**.

### 3.2 Fully automated intervention delivery

#### 3.2.1 Active comparator

In 2018, Allen and co-authors published the results of a three-arm randomized controlled trial of internet-based exercise training (IBET) and in-person physical therapy (PT) versus wait list control [28]. Participants in the IBET arm were encouraged to use the program for 12 months, while those in the PT arm were offered 8 physical therapy visits over 4 months. The IBET program included a personalized exercise regimen using an algorithm based on an individual's responses to a questionnaire. The authors found that IBET was non-inferior to traditional PT at all time points, using a non-inferiority margin of 5 points in total WOMAC score (0-96). In testing the superiority hypotheses, the between group differences in improvement in WOMAC pain scores between the IBET and PT groups were not clinically meaningful at 4-month (−0.47, [−1.2 to 0.26]) and 12-month (−0.45, [−1.18 to 0.27]) follow-up. None of the three groups exhibited clinically meaningful improvement in WOMAC pain from baseline at four- or 12-month follow-up. Neither the IBET and PT groups showed clinically meaningful between group differences in WOMAC pain score improvement from baseline compared to wait list controls at four months (IBET: −0.93 points, [95% CI -1.82 to −0.03]; PT: −0.45, [−1.33 to 0.42]) and improvement in WOMAC pain from baseline to 12 months (IBET: −0.51, [−1.39 to 0.38]; PT: −0.05, [−0.92 to 0.81]) post-randomization.

### 3.3 Intervention includes some telephone or virtual contact from providers

#### 3.3.1 Inactive comparator

Bennell and colleagues tested the effectiveness of internet-delivered exercise and pain coping skills training in a population with chronic knee pain through a randomized trial in 2017 [24]. The authors randomized 148 participants recruited from the community to receive the intervention or educational material accessible through the internet. The intervention spanned 3 months, consisting of educational materials, 7 video sessions with a physical therapist, and pain coping skills training delivered through the internet. The primary outcome was pain during walking (measured on an 11-point Numerical Rating Scale). Those in the intervention group reported significantly improved pain during walking compared to controls at 3 months (between group difference, 1.6 units [95% CI, 0.9–2.3 units]) and 9 months (between group difference, 1.1 units [95% CI, 0.4–1.8 units]). These between-group differences did not meet the MCID threshold. The intervention group improved an average of 2.7 points at 3 months and 2.5 points at 9 months from baseline, which exceeds the minimum clinically important difference (2 points) in NRS pain. Secondary outcomes included WOMAC pain at 3 and 9 months. The intervention group experienced greater improvement in WOMAC pain at 3 months than the controls, and this between group difference in improvement was clinically meaningful. The intervention group experienced a clinically meaningful improvement from baseline in WOMAC pain at all timepoints. Changes in pain scores from baseline to 3- and 9-month follow up in the control group did not meet the MCID.

O'Brien and co-authors conducted a RCT to determine the effectiveness of telephone weight loss coaching compared to usual care in reducing knee pain in knee osteoarthritis patients who were overweight or obese [27]. 120 participants were randomized to receive access to a telephone-based 6-month weight management program or usual care. The primary outcome was knee pain intensity, measured on a Numerical Rating Scale, at every follow-up time point in each group. WOMAC pain was a secondary outcome. The investigators did not report between group differences in pain improvement from baseline to 6 months for either outcome. Neither group experienced a clinically meaningful difference from baseline in NRS or WOMAC pain at 6 months.

Hinman and colleagues conducted a randomized controlled trial of 175 participants to test the effectiveness of a physical therapist-led telephone-delivered exercise and support intervention for patients with knee OA [29]. The control arm received access to an existing service, the Musculoskeletal Help Line, where nurses provide information about OA including treatments, self-management techniques, and assistance navigating services. Those randomized to the intervention arm received access to the same Help Line, as well as 5–10 telephone consultations with a physical therapist over a 6-month period, during which they were prescribed an exercise program. Between group differences in improvement in pain scores were not clinically meaningful at either 6 or 12 months for any pain outcome. Those in the intervention arm experienced a clinically meaningful improvement (at least 2 points) in NRS knee pain from baseline to 6 months (−2.5 points, standard deviation (SD) ​= ​2.0) and from baseline to 12 months (−2.1 points, SD ​= ​2.2) as well as in WOMAC pain and NRS pain on walking at 6 months and 12 months. In contrast, the control group did not experience clinically important improvements for any pain outcomes.

Gohir and colleagues evaluated the Joint Academy program for knee OA in an RCT [25]. Joint Academy is a 6-week online program that provides access to an online physical therapist, exercises, and education about OA through a smartphone application. Those in the intervention arm gained access to Joint Academy and were able to speak to physical therapists about their management plan, which was created based on their questionnaire responses. The control group received usual management for their osteoarthritis symptoms as delivered by their primary care team. At 6 weeks, the intervention group experienced a significantly larger reduction in NRS pain than the control group (between-group difference, −1.5 [95% CI, −2.2 to −0.8]), as well as in WOMAC pain (between-group difference, −1.1 [95% CI, −2.0 to −0.2]), but these between group differences in the extent of pain improvement were not clinically meaningful. The intervention group did not experience a clinically meaningful change from baseline in NRS pain (−1.8 points [95% CI, −2.4 to −1.3]) or WOMAC pain (−2.2 points [95% CI, −2.9 to −1.6]).

### 3.4 Intervention includes some in-person contact with providers

#### 3.4.1 Active comparator

Bennell and co-authors evaluated the use of telephone coaching in addition to an at-home exercise program for 168 subjects with knee osteoarthritis [30]. All participants received five in-person physical therapy sessions over 6 months, were instructed to perform the exercises at home three times per week, and to continue for 12 months after the sessions completed. Half of the participants were randomized to receive an additional 6–12 telephone coaching sessions with a clinician trained in behavior-change support. The primary pain outcome was average knee pain intensity, measured using a Numerical Rating Scale. The change in NRS pain did not differ significantly or meaningfully between groups at 6, 12, or 18 months. Both groups experienced a clinically meaningful change in pain from baseline at 6-month follow up (intervention: 2.4 points [95% CI 1.8 to 2.9]; comparator: 2.0 points [95% CI 1.4 to 2.6]), while only the intervention group's change from baseline met the MCID at 12 months (2.3 points [95% CI 1.7 to 3.0]) and 18 months (2.0 points [95% CI 1.4 to 2.7]). Secondary pain outcomes were NRS pain on walking and WOMAC pain at 6, 12, and 18 months. Differences in change from baseline between groups did not reach the MCID, but the intervention group did experience clinically meaningful changes from baseline.

Baker et al. published the results of an RCT evaluating the effectiveness of the Boston Overcoming Osteoarthritis through Strength Training Telephone-Linked Communication (BOOST-TLC) program in 104 patients with knee osteoarthritis in 2020 [23]. After an initial 6-week exercise class prescribing a strength training routine, participants were randomized to receive the BOOST-TLC program or the control group, both of which received monthly reminders to continue strength training. The BOOST-TLC program consisted of bi-weekly telephone calls to address common barriers to exercise adherence through goal setting, social cognitive theory, counseling messages, and increasing self-effectiveness over a period of 24 months. Participants came for in-person follow up visits at 12 and 24 months to assess outcomes. Pain was a secondary outcome, measured using the WOMAC pain subscale. There were no clinically relevant differences in improvement in pain from baseline to 24-months between groups (between group difference −0.19 points [95% CI -1.80 to 1.42]). Neither group experienced a clinically meaningful improvement in WOMAC pain at 24 months.

#### 3.4.2 Inactive comparator

Hinge Health is a 12-week home intervention with sensor-guided exercise therapy, education, cognitive behavioral therapy modules, and psychosocial support from personal health coaches. After a cohort study on Hinge Health, the authors designed a separate randomized controlled trial to test the benefits of using the program [26,31]. They analyzed 155 participants with chronic knee pain, who were randomized to participate in either the 12-week Hinge Health program or to receive three articles on self-management of chronic knee pain. The primary pain outcome was KOOS pain. Intent-to-treat (ITT) analyses showed that those in the intervention arm had a significant reduction in KOOS pain (between group difference 7.7 points, [95% CI 3.0 to 12.3]) compared to controls at the end of the program, but this difference did not reach the MCID. Similarly, the secondary outcome of VAS pain (between group difference 12.3 points, [95% CI 5.4 to 19.1]) favored the intervention arm compared to the control group but did not reach the MCID. Neither group experienced a clinically meaningful reduction in KOOS or VAS pain from baseline.

Chen et al. [32] evaluated a 12-week home-based exercise intervention, which included 4 in-person health education and exercise group sessions, home exercises, and 5 telephone calls to discuss their home exercises. They analyzed 141 participants who were randomized to either the intervention group (71 participants) or a control group (70 participants), who received the same number of sessions and telephone calls but did not include any exercise-related discussion. WOMAC pain was a primary outcome measured at baseline and 12 weeks. They did not find a clinically meaningful between group difference in change from baseline (−1.6 ​pts [−2.75, −0.58]). We calculated changes from baseline at 12 weeks from mean scores at each timepoint; the intervention group demonstrated a clinically meaningful improvement in WOMAC pain at 12 weeks, while the control group did not.

Li et al. reported the results of an RCT examining the effects of a multifaceted activity intervention, consisting of one in-person group education and individual counseling session (50 ​min total), use of a Fitbit wristband, and PT counseling by phone every other week for 12 weeks [33]. 51 participants were randomized to receive the intervention immediately (26 participants) or be placed into a 13-week waiting period and start the intervention in week 14 (25 participants). KOOS pain, a secondary outcome, was measured at baseline, 13 weeks, 26 weeks, and 39 weeks. There was no clinically meaningful difference in change from baseline to 13 weeks between the two groups (between group difference, 2.5 ​pts [95% CI, −4.2 to 9.5]). Changes from baseline at each timepoint, which we calculated from mean scores at follow-up and baseline, show slight improvement in the control group but not the intervention group, with none reaching the MCID (Table 3).

**Table 3:** Summary of included studies.

Intervention type	Author, Year, Journal	Sample size (# subjects in intervention group)	Avg. age	% female	Trial design, Primary outcome	Intervention summary	Duration of intervention (months)	Pain outcome	Length of follow up (mo)	Change from baseline in intervention group (95% CI), [SD]	Change from baseline in control group (95% CI), [SD]	Between group difference, (95% CI)
***Fully automated intervention delivery***	Allen, 2018, *Osteoarthritis Cartilage*	350 (142)	65.3	72%	RCT, S & N, WOMAC total	Intervention: Program-generated tailored exercises (with video demonstrations) based on pain/function, exercise progression suggestions, automated reminders, progress tracking	12	WOMAC pain 0-20	4	−1.59 (−2.15, −1.02)^b^	PT group: −1.11 (−1.65, −0.58)^b^	WL vs PT: −0.45 (−1.33, 0.42) favoring PT
											WL group: −0.66 (−1.41, 0.09)	WL vs IBET: −0.93 (−1.82, −0.03)^b^ favoring IBET
												IBET vs PT: −0.47 (−1.20, 0.26) favoring IBET
						PT group: up to 8 1-h sessions of PT			12	−1.15 (−1.71 to −0.59)^b^	PT group: −0.7 (−1.23 to −0.16)^b^	WL vs PT: −0.05 (−0.92, 0.81) favoring PT
						WL group: wait list control					WL group: −0.64 (−1.38 to 0.09)	WL vs IBET: −0.51 (−1.39, 0.38) favoring IBET
												IBET vs PT: −0.45 (−1.18, 0.27) favoring IBET
***Intervention includes some telephone or virtual contact from providers***	Bennell, 2017, *Ann Internal Medicine*	148 (74)	61.2	56%	RCT, S, NRS pain on walking	Intervention: Online educational material about OA, automated painCOACH program for pain coping, 7 skype sessions with a physical therapist over 12 weeks	3	WOMAC pain 0-20	3	−3.9 (−3.1 to −4.7)^a^^b^	−1.5 (−0.7 to −2.3)^b^	2.5 (1.5–3.5)^a^^b^ favoring intervention
									9	−3.7 (−2.9 to −4.5)^a^^b^	−2.3 (−1.4 to −3.1)^b^	1.6 (0.6–2.6)^b^ favoring intervention
						Control: Online educational material only		NRS pain on walking 0-10	3	−2.7 (−2.2 to −3.2)^a^^b^	−1.2 (−0.7 to −1.6)^b^	1.6 (0.9–2.3)^b^ favoring intervention
									9	−2.5 (−2.0 to −3.0)^a^^b^	−1.5 (−0.9 to −2.1)^b^	1.1 (0.4–1.8)^b^ favoring intervention
	O'Brien, 2018, Osteoarthritis Cartilage	119 (59)	61.6	62%	RCT, S, NRS pain	Intervention: One telephone call with advice and education about weight loss and physical activity, access to Get Health Information Coaching (10 calls over 6 months)	6	NRS 0-10	1.5	−0.6 c	−0.5 c	Not reported
									6	−0.3 c	−0.9 c	Not reported
						Control: Wait list for orthopedic consultation, usual care provided		WOMAC pain 0-20	1.5	0.2 c	0 c	Not reported
									6	0.5 c	−0.3 c	Not reported
	Hinman, 2020, *Br J Sports Med*	175 (87)	62.5	63%	RCT, S, NRS pain, WOMAC function	Intervention: Access to the Musculoskeletal Help Line and 5–10 telephone consultations with a physical therapist over 6 months	6	WOMAC pain 0-20	6	−3.0 [2.5]^a^	−1.7 [3.1]	1.2 (0.2–2.1)^b^ favoring intervention
									12	−2.9 [2.9]^a^	−2.0 [3.0]	0.8 (−0.2 to 1.7) favoring intervention
						Control: Access to the Musculoskeletal Help Line only		NRS pain overall 0-10	6	−2.5 [2.0]^a^	−1.9 [2.3]	0.7 (0.0–1.4) favoring intervention
									12	−2.1 [2.2]^a^	−1.8 [2.4]	0.3 (−0.4 to 1.0) favoring intervention
								NRS pain on walking 0-10	6	−2.3 [2.5]^a^	−1.2 [2.6]	1.0 (0.1–1.8)^b^ favoring intervention
									12	−2.0 [2.3]^a^	−1.7 [2.4]	0.2 (−0.6 to 1.1) favoring intervention
	Gohir, 2021, *JAMA Network Open*	105 (48)	66.7	68%	RCT, S, NRS pain	Intervention: Access to Joint Academy via mobile app and asynchronous remote communication with physiotherapists	1.5	NRS 0-10	1.5	−1.8 (−2.4, −1.3)^b^	−0.3 (−0.8, 0.2)	−1.5 (−2.2, −0.8)^b^ favoring intervention
						Control: Educational materials, usual care provided		WOMAC pain 0-20	1.5	−2.2(-2.9, −1.6)^b^	−1.2 (−1.8, −0.5)^b^	−1.1 (−2.0, −0.2)^b^ favoring intervention
***Intervention includes some in person contact with providers***	Bennell, 2017, *Arthritis Care Research*	168 (84)	62.3	63%	RCT, S, NRS pain, WOMAC function	Intervention: In person physical therapy followed by a home-exercise program and telephone coaching	6	NRS pain 0-10	6	2.4 (1.8–2.9)^a^^b^	2.0 (1.4–2.6)^a^^b^	0.4 (−0.4 to 1.3) favoring intervention
						Control: In person physical therapy followed by a home-exercise program			12	2.3 (1.7, 3.0)^a^^b^	1.9 (1.3, 2.5)^b^	0.6 (−0.3, 1.5) favoring intervention
									18	2.0 (1.4–2.7)^a^^b^	1.5 (0.8, 2.2)^b^	0.7 (−0.2 to 1.5) favoring intervention
								WOMAC pain 0-20	6	3.5 (2.6–4.3)^a^^b^	2.9 (1.9–3.9)^a^^b^	0.8 (−0.5 to 2.0) favoring intervention
									12	3.2 (2.0, 4.4)^a^^b^	2.1 (0.6, 3.5)^b^	1.5 (−0.3, 3.4) favoring intervention
									18	3.5 (2.6–4.5)^a^^b^	3.7 (2.3–5.0)^a^^b^	0.2 (−1.4 to 1.8) favoring intervention
								NRS pain on walking 0-10	6	2.8 (2.2–3.4)^a^^b^	2.6 (1.9–3.2)^a^^b^	0.2 (−0.7 to 1.0) favoring intervention
									12	2.6 (2.0, 3.2)^a^^b^	1.8 (1.1, 2.5)^b^	0.7 (−0.2, 1.6) favoring intervention
									18	2.5 (1.9–3.2)^a^^b^	2.0 (1.2–2.8)^a^^b^	0.4 (−0.5 to 1.4) favoring intervention
	Baker, 2020, *Arthritis Care Research*	104 (52)	65	81%	RCT, S, exercise adherence	Intervention: In-person exercise program for 6 weeks, followed by strength-training home exercise program, monthly automated reminders, and telephone coaching (weekly for first 6 months, monthly for subsequent 18 months)	24	WOMAC pain 0-20	24	−0.8 c	−0.62 c	−0.19 (−1.80, 1.42) favoring intervention
						Control: In-person exercise program for 6 weeks, followed by strength-training home exercise program and monthly automated reminders						
	Mecklenburg, 2018, *J Med Internet Res*	155 (101)	46	37%	RCT, S, KOOS pain & KOOS physical function	Intervention: One in-person onboarding session, 30 ​min coaching call, access to Hinge Health app	3	KOOS pain 0-100 (0 is best)	3	−10.7 c	−3 c	−7.7 (−12.3, −3)^b^ favoring intervention
						Control: Educational articles only		VAS 0-100	3	−18.6 c	−6.4 c	−12.3 (−19.1, −5.4)^b^ favoring intervention
	Chen, 2019, *BMC Musculoskeletal Disord*	141 (71)	68	84.4%	RCT, S, WOMAC Pain	Intervention: Four in-person 2-h group sessions with physical therapists over 12 weeks, personalized home-exercise program with telephone coaching (every 2 weeks), health education	3	WOMAC pain 0-20	3	−3.06 ac	−1.46 c	−1.60 (−2.75, −0.58)^b^ favoring intervention
						Control: health education only						
	Li, 2020, *JMIR Mhealth Uhealth*	51 (26)	64.9	82%	RCT, S, Time in physical activity	Intervention: One in-person session with group education and PT counseling, Fitbit Flex-2 wristband, biweekly telephone coaching	3	KOOS pain 0-100 (100 is best)	3	0.5 c	0.8 c	2.5 (−4.2, 9.5) favoring control
						Control: Monthly emails of arthritis news unrelated to physical activity			6	−0.1 c	9.7 c	not reported
									9	−0.5 c	7.7 c	not reported
	Allen, 2021, *Ann Internal Medicine*	345 (230)	60	15%	RCT, S, WOMAC total	Intervention: If participant does not meet response criteria, move to the next step	9	WOMAC pain 0-20	3	−1.0 (−1.5, −0.5)^b^	−0.1 (−0.7, 0.5)	−0.9 (−1.7, −0.1)^b^ favoring intervention
						1. Progressive internet-based exercise training (IBET)			6	−1.2 (−1.8, −0.6)^b^	−0.7 (−1.5, 0.0)	−0.5 (−1.4, 0.5) favoring intervention
						2. 6 biweekly telephone coaching sessions			9	−1.0 (−1.5 to −0.5)^b^	0.4 (−0.3 to 1.1)	−1.4 (−2.3 to −0.6)^b^ favoring intervention
						3. In person physical therapy visits						
						Control: Educational materials in the mail every 2 weeks for 9 months						

S ​= ​superiority hypothesis tested, N ​= ​non-inferiority hypothesis tested.

a meets MCID.

b statistically significant.

c mean change from baseline not reported; calculated from mean scores at follow-up and baseline.

Allen and co-authors designed an RCT of a stepped exercise program for patients with knee osteoarthritis (STEP-KOA) [22]. 345 participants from United States Veterans Affairs sites were assigned in a 2:1 ratio to receive either the STEP-KOA intervention or arthritis education control. The STEP-KOA first began with 3 months of an internet-based exercise program. Those who did not exhibit clinically relevant improvement progressed to step 2, which involved 3 months of biweekly phone calls from a physical activity coach. Those who again did not show clinically relevant improvement during step 2 progressed to step 3, which included in-person physical therapy visits. The education control group were mailed educational materials every 2 weeks for 9 months. Pain was measured using the WOMAC pain subscale at 9 months. Between group differences in pain improvement at each timepoint did not reach the MCID (3-month: −0.9 points [95% CI -1.7 to −0.1]; 6-month: −0.5 points [95% CI -1.4 to 0.5]; 9-month: −1.4 points [95% CI -2.3 to −0.6]). Neither group experienced clinically meaningful changes in WOMAC pain from baseline at 3-, 6-, or 9-month follow-up.

## 4 Discussion

This systematic review examined the pain relief offered by remote knee OA self-management programs, with a focus on clinically meaningful changes. In a health landscape increasingly turning to remote care, and especially in view of the COVID-19 pandemic, studies examining the effectiveness of remote OA self-management programs have increased relevance and importance for revamping of clinical care models [34]. Remote care is often more convenient and less expensive for patients: saving time, transportation, and productivity costs without the need to physically be in the physical therapy clinic. Therefore, it is important to understand how patients with knee osteoarthritis may respond to and benefit from remote self-management programs. There are several different treatments for knee OA and a 2021 review of these treatments compared the standardized mean differences (SMD) in pain score between different treatments. For reference, structured exercise programs had a SMD of 0.52 points (95% CI 0.37 to 0.68), compared to corticosteroid injections SMD 0.41 (95% CI 0.21 to 0.61) [2].

We identified eleven RCTs of good quality on the PEDro scale that examined the pain effectiveness of exercise programs with telemedicine components on pain relief for patients with knee OA. For this type of study it can be difficult to blind subjects and assessors to treatment allocation, therefore the maximum PEDro score is often 8 out of 10 points. Three exercise programs administered with telemedicine components provided greater clinically relevant pain relief than in-person physical therapy or waitlist controls. The only trial with a non-inferiority design found their remote program to be non-inferior to in-person physical therapy [28]. These findings align with prior reviews, which found that telemedicine interventions can result in small to moderate improvements in pain [[8], [9], [10]]. One of these recent meta-analyses found significant improvements in function but not pain, suggesting that changes in pain may be less sensitive to intervention effects than function [10].

Only one trial reported a between group difference in improvement from baseline that met an MCID threshold [24]. The control group in this trial only received educational material about knee OA online, while the intervention group received pain coping training and videoconferencing sessions with a physical therapist. The majority of trials in this review (9 out of 10) reported at least one between group difference in pain that favored the intervention group but did not necessarily meet the MCID. Various methods were used as comparator interventions, ranging from wait list controls to groups that received in person physical therapy. Such heterogeneity in comparator groups makes it difficult to compare effectiveness of interventions across studies.

In four trials, the intervention groups experienced clinically meaningful improvements in pain from baseline [24,29,30,32]. In Chen et al., it is difficult to parse the effect of telemedicine treatment, as the controls experienced similar in-person and telemedicine contacts, but without the exercise portion of the intervention [32]. All four interventions included study-initiated follow-up with interventionists over the course of the intervention (over the phone, through videoconferencing, and/or in person), as opposed to subject-initiated online chats or calls. Three contained some element of behavior change counseling (either from a physical therapist or an automated program) [24,29,30]. Four other interventions with similar components did not show clinically meaningful pain improvements from baseline, potentially due to lower adherence to home exercise or intervention calls, and/or not being powered to detect changes in pain as a secondary outcome [22,23,27,33]. Three additional studies reported that their interventions resulted in statistically significant reductions in pain. However, these pain improvements did not reach MCID thresholds, underscoring that statistically significant differences are not necessarily clinically important [22,25,26].

The three studies that used an active comparator (e.g., in person physical therapy) reported similar improvements in pain in both the intervention and comparator groups. One of these programs was fully automated and neither the intervention nor comparator group experienced clinically meaningful reductions in pain [28]. Two programs tested similar interventions (in-person physical therapy followed by telephone coaching) and observed different reductions in pain. The study by Bennell et al. that observed clinically meaningful differences in pain from baseline had an in-person component that spanned 6 months, followed by 12 months of telephone coaching [30]. Baker et al. tested a program with an in-person component that lasted 6 weeks, and only reported pain at 24 months, which did not differ meaningfully from baseline [23]. It is difficult to conclude that a longer in-person component may result in meaningful reductions in pain because Baker et al. did not report pain outcomes earlier than 24 months after randomization and Bennell et al. did not follow-up for 24 months.

This review is limited by the variability of program content, comparators, and outcome measures. Given this, we did not perform a meta-analysis. Additionally, not all trials included pain as a primary outcome, and therefore were not powered to see differences in pain scores. Standard use of outcome measures such as KOOS or WOMAC subscales would be useful for drawing comparisons between future studies and performing meta-analysis. Additionally, there is no universally accepted MCID for any given outcome measure [35].

Studies that used an active comparator suggested that remote exercise programs were not less effective than in-person physical therapy. Studies with an inactive comparator had mixed results, with some programs providing clinically meaningful benefit over controls. Future trials should consider testing interventions that include provider-initiated communications to discuss and personalize exercise programs and incorporate standard comparators (in-person physical therapy and waitlist control) and outcome measures (WOMAC and KOOS). Additionally, no trial has been performed comparing two different forms of remote management in knee OA patients, and we believe a study of this kind would be of value in assessing which intervention elements are most efficacious. When designing future trials, researchers should consider the feasibility of incorporating an intervention into current standard practice on a large scale. Given the potential cost- and time-saving benefits of remote management programs for knee osteoarthritis, such studies would inform clinical and policy decisions.

## Funding

This review was supported by 10.13039/100000069NIAMS grants P30AR072577 and R01AR074290.

## Registration

This systematic review protocol was registered in the PROSPERO database (CRD42022299541).

## Declaration of competing interest

The authors declared no conflicts of interest.
