# Supplementary material for: Effectiveness of remote exercise programs in reducing pain for patients with knee osteoarthritis: A systematic review of randomized trials
Source: Osteoarthr Cartil Open. 2022 May 14;4(3):100264. doi: 10.1016/j.ocarto.2022.100264 (PMC9718080; doi:10.1016/j.ocarto.2022.100264)
Supplement: Multimedia component 1 [file mmc1.docx]

**Appendix 1**

**PubMed Search Term**

“("Osteoarthritis, Knee"[Mesh] OR (("Osteoarthritis"[Mesh:noexp] OR osteoarthrit*[tiab]) AND ("Knee Joint"[Mesh] OR "Knee"[mesh] OR knee[tiab] OR knees[tiab]))) AND ("Telemedicine"[mesh] OR "Mobile Applications"[Mesh] OR "Computers, Handheld"[Mesh] OR telemedicine[tiab] OR telerehab*[tiab] OR telehealth[tiab] OR tele-health[tiab] OR mobile health[tiab] OR mhealth[tiab] OR tele rehab*[tiab] OR e-exercise[tiab] OR telephone delivered[tiab] OR telephone coach*[tiab] OR telephone counsel*[tiab] OR digital care[tiab] OR internet deliver*[tiab] OR internet-based[tiab] OR web-based[tiab] OR digital[tiab] OR text messag*[tiab] OR sms[tiab] OR smartphone*[tiab] OR mobile app*[tiab] OR handheld computer*[tiab] OR handheld device*[tiab]) NOT ("Anterior Cruciate Ligament Injuries" [Mesh], "Anterior Cruciate Ligament Reconstruction" [Mesh])”

Filtered for date range (January 1^st^, 2013 – March 31^st^, 2021) and Clinical Study, Controlled Clinical Trial, Meta-Analysis, Multicenter Study, Observational Study, Randomized Controlled Trial, Review, Systematic Review, Humans

**Embase search term**

("knee osteoarthritis"/exp OR (("osteoarthritis"/de OR "osteoarthrit*":ti,ab,kw) AND ("knee"/exp OR "knee":ti,ab,kw OR "knees":ti,ab,kw))) AND ("telemedicine"/exp OR "Mobile Applications"/exp OR "personal digital assistant"/exp OR "telemedicine":ti,ab,kw OR "telerehab*":ti,ab,kw OR "telehealth":ti,ab,kw OR "tele-health":ti,ab,kw OR "mobile health":ti,ab,kw OR "mhealth":ti,ab,kw OR "tele rehab*":ti,ab,kw OR "e-exercise":ti,ab,kw OR "telephone delivered":ti,ab,kw OR "telephone coach*":ti,ab,kw OR "telephone counsel*":ti,ab,kw OR "digital care":ti,ab,kw OR "internet deliver*":ti,ab,kw OR "internet-based":ti,ab,kw OR "web-based":ti,ab,kw OR "digital":ti,ab,kw OR "text messag*":ti,ab,kw OR "sms":ti,ab,kw OR "smartphone*":ti,ab,kw OR "mobile app*":ti,ab,kw OR "handheld computer*":ti,ab,kw OR "handheld device*":ti,ab,kw) NOT "Anterior Cruciate Ligament Injury"/exp NOT "Anterior Cruciate Ligament Reconstruction"/exp

Filtered for date range (January 1^st^, 2013 – March 31^st^, 2021) and Humans, Cochrane Review, Systematic Review, MetaAnalysis, Controlled Clinical Trial, Randomized Clinical Trial, Article, Article in Press, Data Papers, Editorial, Review, Preprint

**CENTRAL search term**

([mh "Osteoarthritis, Knee"] OR (([mh ^Osteoarthritis] OR (osteoarthrit*):ti,ab,kw) AND ([mh Knee] OR (knee):ti,ab,kw OR (knees):ti,ab,kw))) AND ([mh Telemedicine] OR [mh "Mobile Applications"] OR [mh "personal digital assistant"] OR telemedicine:ti,ab,kw OR telerehab*:ti,ab,kw OR telehealth:ti,ab,kw OR tele-health:ti,ab,kw OR "mobile health":ti,ab,kw OR "mhealth":ti,ab,kw OR "tele rehab*":ti,ab,kw OR "e-exercise":ti,ab,kw OR "telephone delivered":ti,ab,kw OR "telephone coach*":ti,ab,kw OR "telephone counsel*":ti,ab,kw OR "digital care":ti,ab,kw OR "internet deliver*":ti,ab,kw OR "internet-based":ti,ab,kw OR "web-based":ti,ab,kw OR "digital":ti,ab,kw OR "text messag*":ti,ab,kw OR "sms":ti,ab,kw OR "smartphone*":ti,ab,kw OR "mobile app*":ti,ab,kw OR "handheld computer*":ti,ab,kw OR "handheld device*":ti,ab,kw) NOT [mh "Anterior Cruciate Ligament Injury"] NOT [mh "Anterior Cruciate Ligament Reconstruction"]

Filtered for Content type: Trials, Cochrane Library Publication date: between Jan 2013 and Mar 2021, and CENTRAL Trials only: Original Publication year: between 2013 and 2021
